# The Dental Student’s Preparatory Education:—An Address*This Address the writer intended to deliver, substantially, at the Commencement exercises of the Dental College of Ohio, in February, 1856, but was prevented by sickness. He now commits it to the press for the benefit of the graduating class, and with the hope that it will be not without interest to the profession.

**Published:** 1856-04

**Authors:** B. P. Aydelott

**Affiliations:** President of the Board of Trustees of the Dental College of Ohio


### The Dental Register

### OF THE WEST.

VOL. IX. APRIL, 1856. NO. 3.

### Origina Essays and Communications.

### THE DENTAL STUDENTS PREPARATORY EDUCATION :—AN ADDRESS.*

BY REV. B. P. AYDELOTT. D.D.,

President of the Board of Trustees of the Dental College of Ohio.

Educated mind governs the world. And it is right that it should do so. They who are manifestly best qualified to direct and ought, to direct; the hapiness of the masses, the safety of the world demand it.

We say *educated* mind performs this important office, because without education the mind is a mere power,—a blind Samson—either slumbering in the prison-house; or pulling down the edifice to the destruction of multitudes and of itself. Ignorance, almost impotent for good, is yet tremendously mischeivous.

But the mind connot be kcpt entirely uneducated. It will learn. Knowledge is a necessity of the individual as well as of society. Neither could exist without it. A society that knows nothing is an imposibility. And an individual who

* This Address the writer intended to deliver, substantially, at the Commencement exercises of the Dental College of Ohio, in February, 1856, but was prevented by sickness. He now commits it to the press for the benefit of the graduating class, and with the hope that it will be not without interest to the profession.

knows nothing, could do nothing, not even breathe long, and so he must perish for lack of knowledge. Go to the most barbarous tribe, or fragment of a tribe, and you will be sure to find among them the rudiments of knowledge and of the arts. They can at least, barb an arrow, or point a spear, or fashion a snow-shoe, or scoop out a canoe, or contrive a snare of some kind, to take the prey that nourishes them. And as society advances upward its progress all along, is merely the result of increasing knowledge, till at last it reach that high point of civilization which is produced and sustained by a knoweledge so large and multifarous that no one mind nor class of minds can compass it, or wield it for practical purposes·

Hence in such communities the field of knowledge is gradually divided and subdivided, as the capacities and dispositions of individuals and the exigencies of society demand. One betakes himself to this mechanic; art, and ano ther to that. Of the various professional and mercantile persuits, and branches of manufacturing industry, each has its followers. And this mind delights in scientific inquiry, and that in the walks of literature, and a third revels in the beautiful creations of music, or of poetry, or of sculpture But not one of these liveth to himself; he is a laborer for society. Society cannot do without him.

And hence society has a vital interest in this question— What ought to be the education of each individual? How can he be best qualified for his place and his duties in the social organization? most fitly trained as a collaborator in the vast process of the world's civilization?

It is to this great question in one of its phases we would respectfully invite your attention this evening. Our theme is the PREPARATORY EDUCATION OF THE STUDENT OF DENTAL SCIENCE. What ought this to be?

But let us here premise that there are two positions we shall assume in all that follows. First, we as a community, have arrived at that elevated point of civilization which demands the division of the medical profession. No one man among us can, with advantage to himself and society, be at the same time a physician, a surgeon, an accoucheur, a pharmaceutist, a dentist—in a word a universal practitioner of the healing art. Hence the great medical family has spontaneously divided itself off into physicians, surgeons, dentists, pharmeceutists, etc. The progress of society demanded this change; and you can no more turn the medical profession back to the state in which it was some forty years ago, when each practitioner of medicine was not only a physician, a surgeon, and a dentist, but his own apothecary also. With equal success might you attempt to roll back again our beautiful Ohio to its humble tributaries.

And secondly, we take it for granted that dentistry is entitled to the rank of a profession; that is, it demands high educational training for its due culture and practice, and with a lofty unselfish aspect it looks mainly to the good of humanity. This thorough intellectual preparation and a spirit of philanthropy that rises above personal interests we take to be the characteristics of a profession as distinguished from a mere trade or money making art.

What then, we ask, should be the preparatory education of those looking forward to the study of dental science? We answer; it ought, standing in such a relation to a profession, to be just that demanded in all other professions. It ought, in one word, to be SCIENTIFIC and LITERARY.

By *scientific* we mean education in mathematics or the exact sciences, together with the application of mathematical principles to the various branches of natural philosophy. Under the term science we include also the moral sciences, such as, ethics or moral philosophy, political philosophy, political economy, history, logic and psychology or the philosophy of the human mind.

By *literature* we mean the classic writers of Greece and Rome together with those of our own language, and the Rhetoric of these languages.

Such a course of study is necessary to develop and train the whole mind: and it is admirably fitted to this purpose. It gives keenness, vigor, and comprehensiveness to the logical faculties; it rectifies and cultivates the moral feelings and affections, and it purifies and elevates the imagination, the taste, and the critical powers generally. But this is simply the college course; it is just that in which all the great philosophers, statesmen, physicians, lawyers, and divines for the last three centures have been formed. And we despair of ever seeing a better system of training. It applies itself to the whole intellectual and moral man, most thoroughly calls out and strengthens all his powers, and best fits him for the investigation and inculcation of truth, and its practical application in all the varied walks of life. We may indeed improve our college course in its text books and methods of study, but we never can with advantage radically modify it, or lay aside any essential part of it.*

Now that such ought to be the preparatory training of the student of dental science we argue:

---

*Every other course of education is defective because *partial*; and therefore it cannot but fail to do for us what a right education does. He for example, who applies himself exclusively to the mathematics however closely, or to literature, or to the moral sciences must necessarily exhibit more or less of weaknss and *one-sidedness* of character, and so go haltingly all his days. Just as the exclusive use of the arms, or feet, or one side of the body might give efficiency to these, but would leave the rest of the system, ever afterwards, not only incapable of use, but a dead waight, a drag.

The college course avoids these evils. It calls out in full strength and symmetrical development all the capacities and susceptibilities of our nature, and thus brings—not a mere fragment of humanity, but the whole man, with the highest possible advantages, into the field of duty and of trial.

Here, however, we ought to notice an error into which too many well meaning persons have fallen. They suppose that it will take less of time and be cheaper to go through college studies under private tuition. But this is a great mistake. Because, an able faculty, each member thereof being devoted to a single department, can do more for their pupils in every respect, and that with more ease to them and in a shorter period than any *general* educator could. And, because, the fees of a college are always less,on account of its endowment, than those which the private instructor mut have in order to live. So that to take the regular college course will be a saving of time, strength, and money,—to say nothing of those many other advantages which the college only can supply.

So obviously true are the foregoing remarks that it is now no more a question with intelligent men, which is best, a private, or a public education?

I. Because his *professional studies and persuits* require it.

To any intelligent observer who looks at the objects of the dental profession it will be manifest at once that a knowledge of chemistry, mathematics, mechanics, pneumatics and other branches of natural philosophy, are requisite for the due accomplishment of these objects.

But again, the patient of the dental surgeon may suffer a great deal, or by proper professional appliances he may be spared much suffering.

Further, a high and delicate regard for the moral feelings of the patient may be manifested, or the contrary.

And once more; the agreeable appearance, even the beauty of the patient may be greatly preserved and promoted, or it may be destroyed, and even frightful deformity induced, depending on the taste, the skill, and dispositions of the practitioner.

Now who is best prepared for the study and persuit of such a profession? The man who has received a thorough moral, literary, and scientific training? He who has the powers of his intellect called out, quickened, and invigorated in the study of the mathematics, natural philosophy, and chemistry? Whose moral feelings and affections have been cultivated and strengthened by faithful study of the great ethical writers? Whose imagination and taste have been disciplined and elevated by classical and æsthetic studies? It is only necessary to ask these questions; they answer themselves. Every intelligent candid mind will at once see and acknowledge that he who comes to the study and practice of the dental profession thus educated is best prepared to master its various branches, to apply these with the highest advantage to his patient, to meet extraordinary and unforseen occurrences, to improve old methods, to strike out new paths of investigation, and to enlarge the boundaries of his profession.

The difference between the well educated dentist and the unlettered practitioner is just that between the intelligent navigator in a stanch, well rigged ship, with a clear steady light in the binacle, and a well adjusted compass before him; and the rude illiterate sailor with none of these advantages and in an ill furnished bark. He can indeed *work*, and he may perchance, at last make his port, but it is only by *following in the wake of others.*

Now with which of these wonld we be willing to trust ourselves? With the intelligent navigator, or the mere seaman? the educated dentist, or the mechanical practitioner of the art? The man who knows what he is about and is prepared for almost any emergency? or the man who has no principles to guide him, who cannot give the *why* or the *wherefore* for anything?

II. The *debt he owes to his profession and to society* demands of the dentist a thorough preparatory education.

To his profession he is bound to give the best efforts of his mind and time in order, if possible, to add some new principle to its science, or new method to its art, or at least so to improve what is already known, as to leave the profession better than he found it.

To society every intelligent right minded dentist will feel himself under obligation to give the benefits of his profession in its highest improvements, and add to these improvements as far as he can in order that he may, prevent, or relieve, or at least, alleviate human suffering, and increase its enjoyments.

But he is manifestly best prepared to discharge these obligations to his profession and to society, who is the best educated. There can be no doubt—to give a single instance in point—there can be no doubt that had all who have hitherto engaged in the practioe of dentistry been well qualified to record and communicate their observations and reflections in a clear, well digested pleasing manner, the benefits and the boundaries of the profession would have been greatly enlarged. Multitudes of important facts, and new and improved methods, and many precious truths have gone down to the grave with their discoverer, just because, for want of scientific and literary education he was either not able to communicate them to the public, or at least, composition to one of his little culture, was so laborous a task that he was not willing to set about it. What losses has dentistry, like all other persuits in life, sustained in this way.

To the man of thorough scientific and literary attainments composition becomes quite an easy, and often an inexpressibly delightful employment. He can, with comparatively little effort, convey his thoughts in a clear, vigorous, and attractive style, so that his readers are not only instructed, but gratified. Let all our practitioners of dentistry be so educated, and how rapidly would the profession be advanced in its science and practical applications, as well as in the respect and grateful appreciation of the world. But we must hasten to our last argument.

III. The dentist *cannot in our day maintain his position in society* without a liberal education.

We live in an age of progress. Every thing is advancing. Never was there a period in the world's history so distinguished by marvellous discoveries,—discoveries capable of the widest and most important applications,—discoveries calculated to achieve a thorough revolution in the social state. The result has been not only the rapid growth of the sciences, and improvement in the arts, but the general enlightenment of the people, and their advancement in wealth, and in all the comforts of life. An industrious mechanic of this day enjoys more of the real conveniencies of life than did a nobleman in Queen Elizibeth's reign. And woe to the profession, or individual who hangs back at such a time! Not to advance is to retrograde. And he who does not keep up with the improve- of the age can win for himself neither its respect nor its patronage.

In looking back over the history of our city, we cannot call to mind more than two dentists practising here thirty years ago, who could maintain their standing now. The community of this day expect greater things of the profession.

If you cannot come forward with a mind well trained by scientific, literary, and professional culture, your claims to the position you would occupy, be assured, will not be conceded to you. The public now demand, and most reasonably too, that the dental surgeon should be a liberally educated gentleman, as well as a skilful practitioner. He must be prepared not only to discharge well the duties of his laboratory and his office, but to sustain himself in the intercourse of educated, elevated, polished society. The world now almost universally acknowledge your claim to a professional standing, and consequently the world require of you those liberal attainments which can alone qualify you for so honorable a position. Either, then, voluntarily give up the high ground now conceded to you, or else manfully prepare yourselves to fulfil its responsibilities.

And consider also, my young friends, how those who have gone before you toiled and struggled to win this high point for you. See to it, therefore, that you show yourselves worthy of it, for in no other way can you maintain it.

Gratitude, then, to your professional predecessors, who have committed to you so precious a deposit; an honorable feeling of what is due to yourselves and your position;—and a just regard to the claims of society and the expectations of the world;—all these call upon you to transmit this precious deposit greatly enriched to those who shall come after you.

Those, we say, who shall come *after* you. Solemn words! What do they imply? That you *then* have done with earth, and all earth's pursuits and honors; that you *then* have been called to that tribunal at which you must give an account of your stewardship. Ponder, I beseech you, my young friends, upon these thoughts. Let them sink deep into your hearts, and surely you will see and feel that, in addition to what we have already urged, you need those high and holy influences, which only the law of God and the Gospel of his grace can supply, to direct and strengthen you in the great and solemnly responsible work before you. Without these heaven-derived influences, graciously proffered to every sincere inquirer, you will certainly fail of the great end of your being; and life to you, if at all reflecting, must often appear a dark riddle, a feverish dream, a perplexing labyrinth of toils to little purpose, abortive schemes, and disappointed hopes, terminating in a dread, unknown future. But in the light of God's word, how glorious the transformation! The whole scene is at once changed, the mystery cleared up. Everything will appear to you in its right position and value, and a plain path open before you to usefulness, honor, and happiness. With motives—pure, invigorating, exalting—thus pressing upon you from all sides, you cannot fail, under God's blessing, to secure to yourselves the high and influential position of *true* scholars, *true* gentlemen, *true* professional men—because in all *true* Christians.

Heaven, in mercy, grant that such, my young friends, may be your aim, your career, your success; and that at last you may, through grace, receive the righteous approval of the the Searcher of hearts—"Well done, good and faithful servants, enter ye into the joy of your Lord!"

